# P-1031. Urine Trouble (Unless You Bundle): A Multifaceted Approach for Addressing Catheter-Associated Urinary Tract Infections and Asymptomatic Bacteriuria

**DOI:** 10.1093/ofid/ofaf695.1227

**Published:** 2026-01-11

**Authors:** Chrystia Johnson

**Affiliations:** JPS Health Network, Fort Worth, TX

## Abstract

**Background:**

Catheter-associated urinary tract infections (CAUTI) are one of the most common healthcare-associated infections in the U.S. and associated with increased mortality. Each day an indwelling urinary catheter (IUC) is in place, the risk of developing bacteriuria increases. Adherence to evidence-based guidelines for insertion and maintenance of IUCs are essential practices.

**Methods:**

A retrospective case-control review of CAUTIs was done to identify trends and opportunities for improvement. We used a collaborative, multidisciplinary approach to review hospital data and evidence-based guidelines through the implementation of a robust Zero CAUTI/CLABSI Committee (ZCC). This group facilitated several improvement strategies.

**Results:**

In 2018, there were 26 CAUTIs with a standardized infection ratio (SIR) of 1.53. We identified 44% of CAUTIs had opportunities with insertion and 44% with maintenance. The investigation tool was developed by the ZCC as a root cause analysis template and led to a reduction in CAUTIs to 21 (SIR 1.16) in 2019 (p=0.347) and 17 (SIR 0.77) in 2020 (p=0.042).

There was a slight increase of CAUTIs during the height of the COVID-19 pandemic in 2021 with 20 CAUTIs (SIR 0.89).

In 2022, intensive re-education on IUC maintenance, along with implementation of an audit tool, led to a reduction of CAUTIs to 17 (SIR 0.79; p=0.614). This unfortunately did not sustain, and we observed another increase of CAUTIs in 2023 to 20 (SIR 1.01).

Data collected during the maintenance audits exposed an opportunity with a low-quality catheter tubing which was rigid and increased the dependent loops. After changing to a more flexible product, dependent loops decreased. We also implemented a robust clinical decision tool to guide clinicians for testing urine in catheterized patients. In 2024, CAUTIs decreased to 12 (SIR 0.64; p=0.041).

Over the 6-year period, the annual number of reported CAUTIs decreased by 53.8% (p=0.011).

**Conclusion:**

A bundled approach can significantly reduce the burden of CAUTIs. Since these infections are multifaceted, we learned that it is not possible to find one opportunity to solve the problem. Rather, it takes time to analyze data after each intervention to facilitate decision making.

**Disclosures:**

All Authors: No reported disclosures

